# Acupuncture techniques for COPD: a systematic review

**DOI:** 10.1186/s12906-020-02899-3

**Published:** 2020-05-06

**Authors:** Carles Fernández-Jané, Jordi Vilaró, Yutong Fei, Congcong Wang, Jianping Liu, Na Huang, Ruyu Xia, Xia Tian, Ruixue Hu, Lingzi Wen, Mingkun Yu, Natàlia Gómara-Toldrà, Mireia Solà-Madurell, Mercè Sitjà-Rabert

**Affiliations:** 1 School of Health Science Blanquerna, Ramon Llull University, Barcelona, Spain; 2 Global Research on Wellbeing (GRoW) Research Group, Ramon Llull University, Padilla 326-332, 08025 Barcelona, Spain; 3 Centre for Evidence-Based Chinese Medicine, Beijing University of Chinese Medicine, Beijing, China; 4 Faculty of Health Science and Welfare, University of Vic, Vic, Spain; 5 Residència Jaume Batlle, Pere Mata Foundation, Barcelona, Spain

**Keywords:** COPD, Acupuncture therapy, Dyspnoea, Quality of life, Systematic review, Meta-analysis

## Abstract

**Background:**

This is the second part of a large spectrum systematic review which aims to identify and assess the evidence for the efficacy of non-pharmacological acupuncture techniques in the treatment of chronic obstructive pulmonary disease (COPD). The results of all techniques except for filiform needle are described in this publication.

**Methods:**

Eleven different databases were screened for randomised controlled trials up to June 2019. Authors in pairs extracted the data and assessed the risk of bias independently. RevMan 5.3 software was used for the meta-analysis.

**Results:**

Thirty-three trials met the inclusion criteria, which involved the follow techniques: AcuTENS (7 trials), moxibustion (11 trials), acupressure (7 trials), ear acupuncture (6 trials), acupressure and ear acupuncture combined (1 trial) and cupping (1 trial). Due to the great heterogeneity, only 7 meta-analysis could be performed (AcuTENS vs sham on quality of life and exercise capacity, acupressure vs no acupressure on quality of life and anxiety and ear acupuncture vs sham on FEV_1_ and FEV_1_/FVC) with only acupressure showing statistical differences for quality of life (SMD: -0.63 95%CI: − 0.88, − 0.39 I^2^ = 0%) and anxiety (HAM-A scale MD:-4.83 95%CI: − 5.71, − 3.94 I^2^ = 0%).

**Conclusions:**

Overall, strong evidence in favour of any technique was not found. Acupressure could be beneficial for dyspnoea, quality of life and anxiety, but this is based on low quality trials.

Further large well-designed randomised control trials are needed to elucidate the possible role of acupuncture techniques in the treatment of COPD.

**Trial registration:**

PROSPERO (identifier: CRD42014015074).

## Introduction

Chronic obstructive pulmonary disease (COPD) is one of the most prevalent lung diseases, with 251 million cases globally in 2016, and is the 4th cause of death worldwide, with more than 3.2 million instances in 2015 [1]. These numbers are expected to increase [2].

COPD is characterised by a chronic and irreversible airflow obstruction caused by an inflammation in the airways and lung parenchyma which leads to structural abnormalities in the airways. These alterations specially affect force expiratory volume in the first second (FEV_1_) compared to force vital capacity (FVC) [3]. The main symptoms of this disease are progressive dyspnoea, chronic cough, sputum production and recurrent respiratory infections. Those symptoms get worse as the disease evolves, with many effects on exercise capacity and quality of life [4].

The usual treatment in COPD targets its main symptoms. Pharmacological treatment includes the use of corticosteroids and bronchodilators to reduce airway inflammation and obstruction, and non-pharmacological treatment such as pulmonary rehabilitation are used to improve perceived dyspnoea, exercise capacity and quality of life [3].

Acupuncture derives from Traditional Chinese Medicine, which uses different techniques to stimulate specific areas of the body surface, or acupuncture points, to restore health. Even though inserting needles is the best-known acupuncture technique (filiform needle acupuncture), there are several others, including heat stimulation (moxibustion), electricity (electroacupuncture or acupoint transcutaneous electrical nerve stimulation (AcuTENS)), and digital pressure (acupressure). These techniques have been traditionally used to treat all kinds of health problems including respiratory diseases like COPD, however there is little evidence about the effectiveness of those techniques and no previous review has studied different acupuncture techniques individually.

The aim of this review is to identify and separately evaluate the efficacy of non-pharmacological acupuncture techniques, excepting via filiform needle. These techniques include moxibustion (except when performed alongside use of an acupuncture needle), electroacupuncture (when not delivered using an acupuncture needle) AcuTENS, acupressure and ear acupuncture, and cupping therapy among others.

## Methods

### Protocol and registration

We followed the recommendations of the Cochrane Handbook for Systematic Reviews of Interventions [5] for this review and the Preferred Reporting Items for Systematic Reviews and Meta-Analyses (PRISMA) statement [6]. The protocol was previously registered at PROSPERO (CRD42014015074) and is available on: http://www.crd.york.ac.uk/PROSPERO/display_record.asp?ID=CRD42014015074).

### Eligibility criteria

We included randomised controlled trials or quasi-randomised trials and crossover trials, meeting all the following criteria: [1] performed in COPD patients with different grades of obstruction (GOLD A to D) in exacerbation or stable periods [2]; assessing non-pharmacological modalities of acupuncture (filiform needle, electroacupuncture, acupressure, moxibustion, ear acupuncture, etc.) compared with a control group (sham acupuncture or no acupuncture), in addition to usual care (medication, physiotherapy, pulmonary rehabilitation, etc.); and [3] reporting at least one of the following outcomes: dyspnoea, quality of life, adverse effects, exercise capacity, lung function or anxiety and depression.

Exclusion criteria were: [1] if acupuncture was compared with a different acupuncture technique or a therapy not used in usual care; and [2] randomised cluster studies.

No language restriction was applied.

Due to the large number of acupuncture techniques found, we decided to exclude those that were mainly used only in China and to focus on those best known and practiced elsewhere in the world. Trials involving the techniques are listed in the results section, but were not analysed. This exclusion criteria was not applied in our original protocol.

### Information sources

An electronic search was performed up to June 2019. The databases included were the Cochrane Central Register of Controlled Trials (CENTRAL), Medline, Embase, CINAHL, AMED (Ovid), PEDro, PsycINFO, CNKI, VIP, Wanfang and Sino-Med. the bibliographies of selected articles were also consulted in search of additional studies not detected in the initial searches. Manual reviews were also performed on international respiratory diseases conferences (European Respiratory Society and American Association for Respiratory Care) from 2010 to 2017.

### Search

We conducted a comprehensive search using the following key words and their variations: “acupuncture”, “moxibustion”, “acupressure”, “electroacupuncture”, “AcuTENS”, “ear acupuncture”, “cupping”, “COPD”, “randomised control trial”. The search strategy was adjusted for each database (see Supplementary material 1).

### Study selection

The reviewers (CFJ, MSR, JV, WC, HN, XRY, TX, HRX MS, NGT) worked in pairs and independently identified the articles that met the inclusion criteria, first through title and abstract and afterwards through full text paper.

### Data collection process

Reviewers (CFJ, MSR, JV, WC, HN, XRY, TX, HRX MS, NGT), both in pairs and independently, extracted data using a standardised data extraction form. A pilot test was performed prior to data extraction to check the suitability of the form, as well as its understanding by the reviewers. A third author was consulted in the case of discrepancies. Lack of data or inconsistent data were managed by contacting trial authors; if this was not possible the data was not included in the meta-analysis.

### Risk of bias in individual studies

The Cochrane Risk of Bias Assessment Tool [7] was used to assess the risk of bias in the papers. Due to the nature of acupuncture techniques, the Cochrane risk of bias tool was modified to add “blinding of outcome assessment”. “Blinding of personnel” was removed because a person providing acupuncture treatment cannot be blinded.

### Summary measures

Continuous outcomes were expressed as mean difference (MD) with 95% confidence interval (CI) or standardised mean difference (Std. MD) when different scales were used. For trials with different arms using acupuncture, the results were combined before meta-analysis using the Cochrane Handbook [8].

### Synthesis of results

The heterogeneity of the studies was evaluated using the I^2^ statistic. Post-treatment data from each group or post treatment differences between groups were used for the meta-analysis. When this was not reported or large baseline differences were found between the groups, the difference from baseline data from each group were used. The results were combined in a meta-analysis using RevMan 5.3 software and applying a fixed effects model to summarise the results when heterogeneity was not relevant (I^2^ < 30%). Otherwise, a random effects model was used. If I^2^ value was over 70%, a narrative synthesis of the available data was performed.

### Additional analyses

Since studies included patients with different conditions (stable and exacerbation) and this could lead to heterogeneity in our results, we decided to separate them into two subgroups in all meta-analyses. The results are therefore presented separately when heterogeneity was too big (I^2^ < 70%) between subgroups or in one of the subgroups.

## Results

### Study selection

**Fig. 1:**
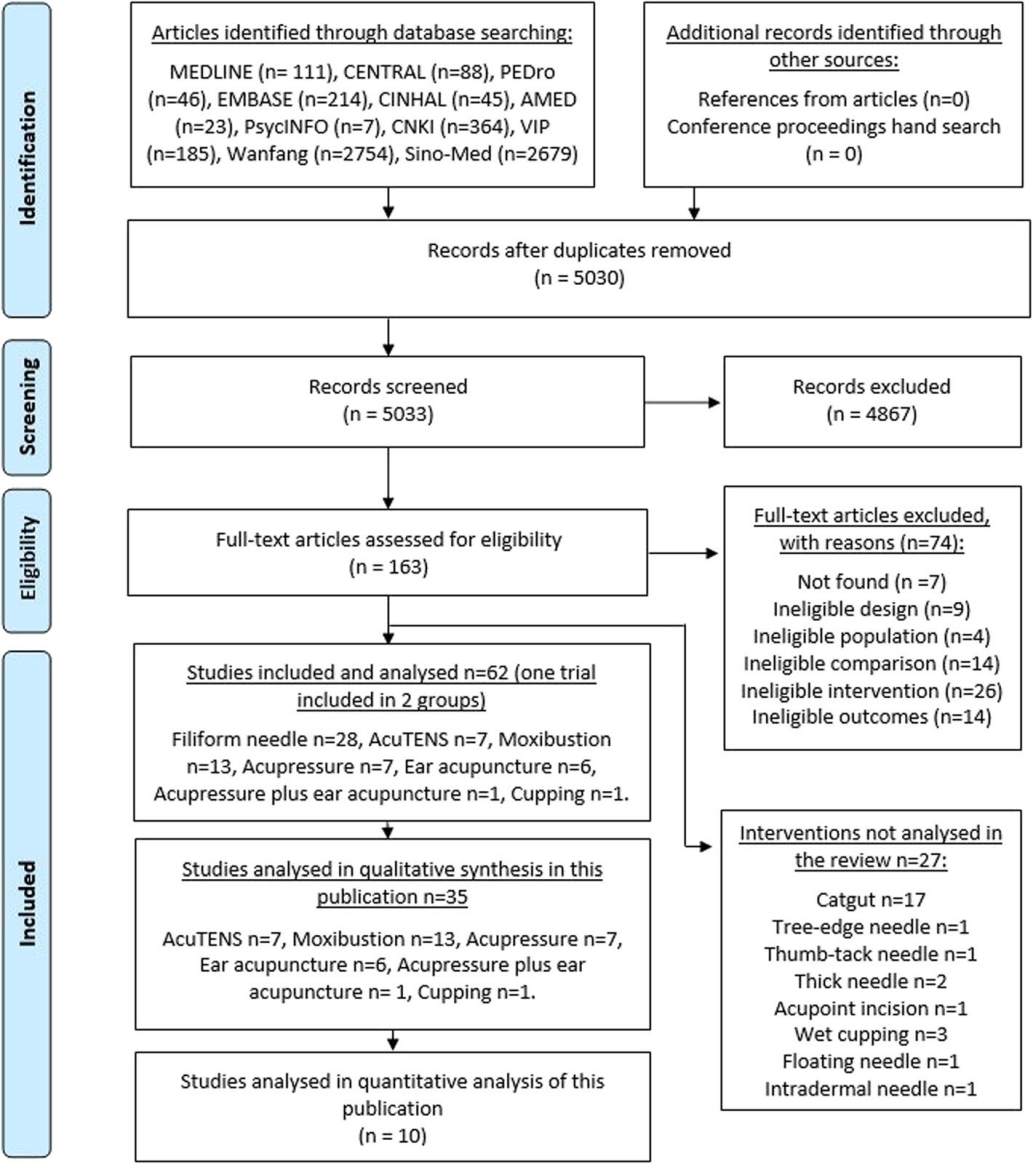
Flow diagram

To identify potentially eligible studies, reviewers in pairs independently screened all 5030 unduplicated titles and abstracts retrieved, and the full text of 163 articles was obtained for decisions about final inclusion. Forty-eight articles were excluded for the reasons shown in Fig. 1. As mentioned in the methods section, several acupuncture techniques used only in China were not included in the analysis: catgut implant (17 studies), tree-edge needle (1study), thumb-tack needle (1 study), thick needle (2 studies), acupoint incision (1 study), wet cupping (3 studies) or floating needle (1 study) and intradermal needle (1 study). Sixty-two studies (36 publications) were included and analysed in the review.

In this publication we included the results from 35 trials (36 publications) which used all other techniques except filiform needle: AcuTENS (7 trials) [9–15], moxibustion (13 trials) [8, 16–27], acupressure (7 trials from 8 publications) [28–35], ear acupuncture (6 trials) [36–41], acupressure combined with ear acupuncture (1 trial) [42] and cupping technique (1 trial) [43] (one trial with multiple arms was included in the filiform needle group and the ear acupuncture group) (Fig. 1).

### Study characteristics

The details of all trials included, classified for intervention, are summarised in Table 1.

**Table 1:** Details from all trials

First author, year	Study design	Subjects analysed (M:F) Severity	Age: Mean (SD)	Intervention (I)	Control (C)	Stimulation time	Treatment regimen	Outcomes
AcuTENS vs. sham AcuTENS								
Stable patients								
Lau, 2008 [9]	RCT	46 (31:15) Mild to moderate	75 (7.0)	AcuTENS at Ding Chuan (EX-B1). Frequency of 4 Hz and pulse width 200 μs. Intensity at the highest tolerable by the participant.	Placebo TENS with no electrical output at same point as treatment group	45 min	Single session	Dyspnoea (SOB VAS) Lung Function (FEV_1_, FVC)
Liu, 2015 [10]	RCT	50 (25:25) Moderate to very severe	66.3 (9.0)	AcuTENS at Ding Chuan (EX-B1), BL13, BL23, ST36 Frequency of 2 Hz + Usual treatment	Placebo TENS with no electrical output at same points as treatment group + Usual treatment	40 min	Every 2 days for 4 weeks (14 sessions)	Dyspnoea (DVAS after 6mwd), QoL (CAT), Exercise capacity (6mwd), Lung function (FEV_1_, FVC)
Ngai, 2010 [11]	RCT	18 Not mentioned	71.8 (1.2)	AcuTENS at Ding Chuan (EX-B1). Frequency of 2 Hz, pulse width of 200 μs.	Placebo TENS with no electrical output at same point as treatment group	45 min	20 sessions. 5 sessions per week for 4 weeks	QoL (SGRQ) Exercise capacity (6mwd) Lung function (FEV_1_, FVC)
Jones, 2011 [12]	RCT	44 (25:19) Not mentioned	69.1 (1.6)	AcuTENS at Ding Chuan (EX-B1). Frequency of 2 Hz, pulse width of 200 μs	Placebo TENS with no electrical output at same point as treatment group	45 min	Single session	Dyspnoea (VAS) Lung function (FEV_1_, FVC,)
Shou, 2014 [14]	RCT	30 (10:20) Mild to moderate	I: 68.3 (10.2) C: 70.0 (9.2)	TENS at bilateral BL13 Frequency of 4 Hz, pulse width of 200 μs	Placebo TENS with no electrical output at same point as treatment group	40 min	20 sessions. Five times a week for 4 weeks	Lung Function (FVC, FEV_1_,)
Wen, 2011 [15]	RCT	40 (14:26) Not mentioned	I: 69.7 (8.09) C: 66.9 (6.71)	TENS at Ding Chuan (EX-B1) Frequency of 4 Hz, pulse width of 200 μs. + conventional treatment	Placebo TENS with no electrical output at same point as treatment group + conventional treatment	40 min	Once a day for 10 days	Lung function (FEV1, FVC)
Exacerbated patients								
Öncü, 2017 [13]	RCT	70 (54:16) Not mentioned	Not mentioned	AcuTENS at Ding Chuan (EX-B1) and LU7. Frequency 4 Hz, pulse width of 200 μs +Conventional treatment	Placebo TENS with no electrical output at same point as treatment group +Conventional treatment	45 min	20 sessions. Daily at hospital and 3 times a week at home	Dyspnoea (mMRC, Borg), QoL (SGRQ) Exercise capacity (6mwd)
Moxibustion vs. no moxibustion								
Stable patients								
Bai, 2018 [26]	RCT	80 (44: 36) Mild to severe	I: 64.6 (5.0) C: 63.7 (5.2)	Moxibustion with a moxa stick at GV14 and CV17 + Routine treatment	Routine treatment	5 min per point	Once a day for 30 days	QoL (SGRQ) Lung Function (FEV_1_,)
Cheng, 2011 [23]	RCT	60 (42:18) Not mentioned	I1: 65.07 I2: 68.15 C: 69. 21	I1: Heat-sensitive point’s moxibustion (gentle moxibustion at heat-sensitive points) I2: Moxa stick. 3–5 points selected depending on symptom. BL12, BL13, BL20, BL23, LU7, LU9, CV12, ST36, SP6, KI3, ST40 + Western medicine standard therapy	Western medicine standard therapy (Anti-inflammatory, relieve panting, eliminating phlegm to stop cough)	I1: Until diathermy disappeared and patients felt burning pain. I2: 30 min	Once daily. 30 days	Lung function (FEV_1_, FEV_1_/FVC)
Cui, 2015 [22]	RCT	60 (34:26) Not mentioned	56 ± 8.1	Moxa sticks at GV14 and GV2 + Routine treatment	Routine treatment (theophylline sustained-release capsules and ambroxol tablets)	Not mentioned	Once a month for 3 months	Lung function (FEV_1_, FEV_1_/FVC,)
Guang, 2017 [25]	RCT	60 (31:29) Not mentioned	I: 56 (1) C: 55 (2)	Moxibustion with 3 moxibustion boxes from GV14 to GV2 + Tiotropium bromide inhalation powder spray	Tiotropium bromide inhalation powder spray	20 min	5 time per week for 12 weeks	Dyspnoea (mMRC) QoL (CAT)
He, 2013 [21]	RCT	93 (63:30) Not mentioned	I: 66.11 (9.34) C: 67.25 (8.75)	Ginger moxibustion at BL13 bilateral. Each time 3 / 5 wicks + Compound methoxamine capsule	Compound methoxamine capsule	Not mentioned	Once every 3 days, a total of 14 times Oral treatment: 3 times a day for 6 weeks	Lung function (FEV_1_, FEV_1_/FVC, FVC)
Liang, 2018 [27]	RCT	88 (51:37) Moderate to severe	I: 65. 69 (7.22) C: 65. 96 (7.19)	Heat sensitivity moxibustion between BL13 and BL17 points +Routine treatment	Routine treatment	5 min per point	5 times a week for 8 weeks	Lung function (FEV_1_, FEV_1_/FVC)
Liu, 2015 [20]	RCT	100 (61:39) Not mentioned	67.5 (9.2)	Moxibustion therapy with moxa stick at GV14, BL13, Ding Chuan (EX-B1), ST40, ST36 +Routine treatment	Routine treatment (low flow oxygen therapy and bronchodilator and antibiotic)	30 min	Once a day for 14 days	QoL (SGRQ), Exercise capacity (6mwd), Lung function (FEV_1_, FVC, FEV_1_/FVC)
Tang, 2012 [19]	RCT	40 (29:11) Not mentioned	I: 75.5 (13) C: 77.8 (2.3)	Moxibustion at BL12, BL20, KI1, ST36, ST40 + Western medicine treatment	Western medicine treatment (continuous low-flow oxygen inhalation, anti-inflammatory, relieving asthma, eliminating phlegm, stopping cough and immune support)	10-15 min	Once a day for 4 weeks	TCM syndrome (cough, phlegm, asthma, full attack time)
Wang, 2016 [18]	RCT	70 (56:14) Not mentioned	I: 65.2 (6.1) C: 66.3 (6.3)	Moxibustion with moxa stick and moxibustion box at RN8, RN6, RN4, BL12, CV12, ST36 +Routine treatment	Routine treatment (oxygen therapy, nutrition support, respiratory rehabilitation)	10–15 min per point	3 treatment courses. Each course consisted in 14 daily consecutive sessions.	Lung function (FEV_1_, FVC)
Wen, 2013 [8]	RCT	108 (67:41) Not mentioned	Not mentioned	Cone Moxibustion at BL13, BL15, BL18, BL20, BL23 Patients feel burning sensation, intolerance, to remove residual wick, replace with a new wick. +Western medicine treatment	Western medicine treatment (spasmolytic, relieving asthma, eliminating phlegm, stopping cough drug treatment)	Not mentioned	5 times a week for 4 weeks	QoL (SGRQ)
Yang, 2016 [17]	RCT	60 (42:18) Not mentioned	54.1 (9.75)	Moxibustion with cones from GV3 to GV14 +Routine treatment	Routine treatment (oxygen inhalation, thiamethoxam bromide, budesonide)	2 h	9 sessions, once every 10 days for 3 months	Lung function (FEV_1_, FEV_1_/FVC)
Zhang, 2016 [16]	RCT	510 (308:202) Not mentioned	62 (9)	Moxibustion with moxa stick at BL13, BL20, GV12, LU1, CV6, ST36, ST40, KI3 +Montelukast	Montelukast (10 mg/day, oral)	5–10 min per point	4 to 6 courses during a year. Each course consisted in 10 daily sessions	Lung function (FEV_1_, FEV_1_/FVC)
Zhe, 2017 [24]	RCT	80 (44:36) Mild to severe	I: 58.2 (11.7) C: 57.5 (12.3)	Moxibustion to 6 to 7 heat sensitive points found between the horizontal lines of BL13 and BL17 + Conventional treatment	Conventional treatment (bronchodilators, glucocorticoids, expectorant cough and respiratory exercises)	30 to 40 min	5 times a week for 3 months	QoL (SGRQ) Lung function (FEV_1_, FEV_1_/FVC)
Acupressure vs. sham acupressure								
Stable patients								
Wu, 2004^a^ [31] 2007^b^ [29]	RCT	44 (36:8) Not mentioned		Effleurage: hold, rub and press the neck and each shoulder Press and rub GV14 3 min. Press the CV22 for 1.5 min. Press and rub the BL13 for 3 min. Press and rub the BL23 for 1.5 min. Press and rub LU10 for 3 min	Effleurage: hold, rub and press the neck and each shoulder Rub and press Sp5 for 4 min. Rub and press Sp3 for 4 min. Point (using finger-tip pressure only) and press Liv1 for 4 min.	16 min	20 sessions. Five times a week for 4 weeks.	Dyspnoea (PFSDQ-M)^a^, (VAS)^b^ Exercise capacity (6mwd)^a^ Anxiety (SSAI)^a^, Depression (GDS)^b^
Maa, 1997 [30]	CRCT	31 (19;12) Not mentioned	67.32 (8.17)	Acupressure at LU1, LU2, LU10, PC8, ST36, LI4, GV14 +Pulmonary rehabilitation	Sham acupressure: pressure to no specified sham points +Pulmonary rehabilitation	1 or 2 min per acupoint	at least once a day for 6 weeks Pulmonary rehabilitation: 21–36 sessions	Dyspnoea (mBorg and VAS) Exercise capacity (6mwd) Anxiety (BESC)
Acupressure vs no acupressure								
Stable patients								
Guo, 2017 [32]	RCT	200 (not mentioned) Not mentioned	Not mentioned	Acupressure at GV14, Ding Chuan (EX-B1), BL23, BL13, BL17, CV12 and CV17 +Regular treatment	Regular treatment (drug treatment and respiratory exercises)	2–3 min per point	Once a day for 6 months	QoL (SGRQ), Anxiety (HAM-A), Depression (HAM-D) Pulmonary function (FEV_1_, FEV_1_/FVC)
Huang, 2018 [33]	RCT	68 (not mentioned) Not mentioned	I: 52.4 (3.9) C: 54.4 (1.2)	Acupressure at BL13, BL20 and GV14 + Routine drug treatment	Routine drug treatment	2 min per point	Twice a day for 3 months	Exercise capacity (6mwd), QoL (CAT), Pulmonary function (FEV_1_, FVC, FEV_1_/FVC)
Wu, 2017 [34]		64 (38:26) Not mentioned	73.6 (6.7)	Acupressure at GV20, GB20, Taiyang, ST36, PC6 and LI11 + Regular treatment	Regular treatment (drug treatment, psychological nursing, health guidance and diet adjustment)	5–10 min per point	Twice a day for 4 weeks	QoL (GQOL - 74)
Xu, 2018 [35]	RCT	98 (51:47) Not mentioned	63.1 (15.2)	Acupressure at CV12, CV4, CV6 + Regular treatment	Regular treatment	10 min per point	Not mentioned	Anxiety (HAM-A, SCL-90)
Exacerbated patients								
Tsay, 2005 [28]	RCT	52 (25:27) Not mentioned	73.88 (7.19)	Acupressure at LI4, PC6 and Ear ShenMen + 3 min shoulders massage + Regular treatment	3 min shoulders massage + Regular treatment (inhaled bronchodilators and mechanical ventilation)	15 min	Once a day for 10 days	Dyspnoea (VAS) Anxiety (SSAI)
Ear acupuncture vs. sham ear acupuncture								
Exacerbated patients								
Cao L, 2012 [36]	RCT	30 Not mentioned	I: 76.9 (5.84) C: 77.6 (5.70)	Ear acupressure with seeds at: Shenmen, Lung, Trachea, Throat, Inter-tragus + Sham acupuncture + Usual treatment	Sham auricular therapy at irrelevant acupoint + Sham acupuncture + Usual treatment (bronchodilator, anti-inflammatory, anti-choline drug)	Pressing the seeds: 3–5 times a day	20 days	Dyspnoea (mMRC) QoL (CAT) Lung Function (FEV_1_, FEV_1_/FVC)
Ear acupuncture vs no ear acupuncture								
Stable patients								
Jin RF 2009 [37]	RCT	60 (39:21) Not mentioned	Not mentioned	Ear acupressure with seed at: Lung, Spleen, Kidney, Trachea, Under sebum, Sympathetic + Regular treatment	Regular treatment (eliminating phlegm, bronchodilator, regular nursing)	Not mentioned	Once a day for 12 days	Lung Function (FVC, FEV_1_, FEV_1_/FVC)
Li 2017 [40]	RCT	82 (not mentioned) Not mentioned	Not mentioned	Acupressure using magnets at: Anti-asthmatic point, Trachea, Lung, Shenmen, Occiput, Adrenal gland +Regular treatment	Regular treatment	Press 1020 times per point	I1: once every 6 h, (at least 3 times a day) for 6 months. I2: at least 3 times a day from 3 am to 5 am and 3 pm to 5 pm for 6 months.	Dyspnea (mMRC) Lung function (FEV_1_, FVC, FEV_1_/FVC)
Pang CL, 2014 [39]	RCT	52 (31:21) Not mentioned	I: 62.5 (6.4) C: 68.2(6.0)	Ear acupressure with seeds at: Spleen, Kidney, Lung and Sanjiao + Inhaled Seretide	Inhaled Seretide.	Not mentioned	Press seeds 3 times a day for 3 months	Lung Function (FEV_1_, FEV_1_/FVC)
Pang CL, 2016 [38]	RCT	38 (25:13) Severe and very severe	I: 65.5 (6.4) C: 67.2(6.3)	Ear acupressure at: Spleen, Kidney, Lung, Sanjiao and Relieving asthma + Salmeterol inhalation powder	Salmeterol inhalation powder	2 min	Massage 3 times a day for 3 months	Lung Function (FEV_1_, FEV_1_/FVC)
Ear acupuncture vs drugs								
Exacerbated patients								
Hu ZH, 1997 [41]	RCT	32 (19:13) Not mentioned	I: 63.5 (12.06) C: 60.33 (12.45)	Ear acupuncture with manual needle stimulation at: Lung, Trachea and Inter-tragus	Inhaled salbutamol	30 min	1 session	Lung function (FEV_1_, FVC,)
Cupping vs no Cupping								
Exacerbated patients								
Xiao W, 2009 [43]	RCT	60 (33:27) Not mentioned	I: 72 (51–81) C: 70 (48–85)	Flash Fire Cupping therapy at BL13, BL20 and BL23 + Western medicine treatment	Western medicine treatment (oxygen inhalation, spasmolytic, relieving asthma, eliminating phlegm, stopping cough treatment)	Not mentioned	28 sessions, once a day for 4 weeks	TCM syndrome integral (cough, expectoration, dyspnoea, wheezing)

*RCT* randomised control trial, *CRCT* Cross-over randomised control trial, *SOB* shortness of breath, *VAS* visual analogue scale, *FEV1* forced expiratory volume in one second, *FVC* forced vital capacity, *QoL* quality of life, *SGRQ* St George’s respiratory questionnaire, *6MWD* 6-min walking distance, *PEFR* peak expiratory flow rate, *RR* respiratory rate, *QoL* quality of life, *CAT* COPD assessment test, *COPD* chronic obstructive pulmonary disease, *PaO2* arterial oxygen partial pressure, *PaCO2* arterial partial pressure of carbon dioxide, *PFSDQ-M* pulmonary functional status and dyspnoea questionnaire, *SSAI* Spielberger’s state anxiety inventory, *VAS* visual analogue scale, *BESC* bronchitis-emphysema symptom checklist, *GDS* geriatric depression scale, *HAM-A* Hamilton anxiety rating scale, *HAM-D* Hamilton depression rating scale, *GQOL–74* generic quality of life inventory-74, *SCL-90* symptom checklist-90

#### Design

All trials were classified as randomised control trials since they all reported that groups were generated randomly, however, 14 trials did not provide sufficient information about the sequence generation process. Only one trial used a cross-over design [30].

#### Participants

The mean age of participants ranged from 52 to 78 years and was similar across all interventions. From the 34 included trials, severity was not reported in 29. Most trials included mild to severe participants. After reading each paper carefully, 29 trials were classified as treating stable patients [8–12, 14–27, 29–34, 37–40] and 6 trials were classified as treating exacerbated patients [12, 27, 35, 40–42].

#### Interventions and comparisons

##### AcuTENS

Seven trials using AcuTENS were included. All trials used a similar protocol that consisted of using a stimulation pulse of between 2 and 4 Hz with a wave width of 200 microseconds and a stimulation time of 40–45 min. Five out of seven papers used a single point treatment, with four papers using Ding Chuan (EX-B1) [9, 11, 12, 15] and one using BL 13 [14]. Only two papers used a combination of several points [10, 13]. The main differences between protocols were seen in the treatment regime, with two papers using a single session intervention [9, 12] whereas in the other five papers stimulation was used from 10 to 20 sessions and a frequency between seven and four sessions a week.

All seven trials were sham-controlled using an AcuTENS device with no electric output.

##### Moxibustion

The thirteen studies included used multiple moxibustion techniques, including moxa stick [16, 18, 20, 22, 23, 26], heat sensitivity moxibustion [23, 24, 27], cone moxibustion [8, 17], moxibustion boxes [25] and ginger moxibustion [19, 21]. There were a number of acupuncture points used, ranging from 1 to 12, but most of the trials used from 3 to 7 points [8, 18–20, 23]. Most common acupuncture points used were.

Bl13 (7 trials, 53%), GV14, BL20 and ST36 (5 trials, 28%). Treatment regimens were also different, with two studies using from 3 to 6 treatment courses of 10–14 consecutive days during a year [16, 18], three studies using daily treatments for a period of 2 to 4 weeks [19, 20, 23], two using from 2 to 5 treatments a week over a month [8, 21], two trials using 5 treatments a week over 3 to 4 months [24, 25], one using nine treatments every 10 days over 3 months [17] and one trial using one treatment per month [22]. In all thirteen papers, moxibustion was added to the usual treatment and compared with the usual treatment alone.

##### Acupressure

Out of the 7 included trials using acupressure, 5 trials included used 5–7 points 26–28, [32, 34] while 2 trials used 3 points [33, 35]. Most common used point were GV14 (4 trials, 57%) and BL13 (3 trials, 43%). Most trials used rubbing and pressing stimulation (1–3 min per point), in 5 to 15 min sessions but differed in the acupoints used, and in the session regime, which ranged from at least one treatment a day to five treatments a week over 4–24 weeks [29–31]. One study differed clearly from the others in using two points of stimulation plus the ear acupuncture point Shenmen in a daily treatment regime for 10 days [28].

Acupressure was compared with sham acupressure in two studies (three publications) using the stimulation of non-specific points [29–31]. Acupressure plus the usual treatment was compared with the usual treatment alone in five trials [28, 32–35].

##### Ear acupuncture

The six studies included used from four to seven ear acupuncture points. Most common points were Lung (6 trials, 100%) and Trachea (4 trials, 66%). Ear acupuncture was performed using seed stimulation [36–38], acupressure stimulation [39], stimulation with magnets [40] or filiform needle stimulation [41]. In four trials [36–39], the intervention duration ranged from 12 days to 6 months and only one trial used a single session treatment [41].

Sham control was used in only one trial using irrelevant ear points [36]. Four trials compared the usual treatment plus ear acupuncture with the usual treatment alone [37–40]. One compared ear acupuncture with inhaled salbutamol [41].

##### Acupressure plus ear acupuncture

One trial combined acupressure plus ear acupuncture added to usual treatment compared with usual treatment alone [42].

##### Cupping

One trial studied the effect of cupping plus usual treatment compared with usual treatment alone [43]. In this trial fire cupping with flash stimulation at BL13, BL20 and BL23 was used for 28 sessions for 4 weeks.

### Risk of bias within studies

An assessment of the risk of bias for the included trials is summarised in Table 2.

**Table 2:** Detailed risk of bias of each trial

	Random sequence generation	Allocation concealment	Blinding of participants	Blinding of outcome assessment	Incomplete outcome data	Selective outcome reporting	Other sources of bias
AcuTENS							
Lau, 2008 [9]	Low	Low	Low	Low	Low	Unclear	Low
Liu X, 2015 [10]	Low	Low	Low	Low	Low	Unclear	Low
Ngai, 2010 [11]	Low	Unclear	Low	Low	Low	Unclear	Low
Jones, 2011 [12]	Low	Unclear	Low	Low	Unclear	High	Unclear
Öncü, 2017 [13]	Low	Unclear	Low	Unclear	Unclear	Unclear	Unclear
Shou, 2014 [14]	Low	Unclear	Unclear	Unclear	Low	Unclear	Unclear
Wen Q 2011 [15]	Low	Unclear	Low	Unclear	Low	Unclear	Unclear
Moxibustion							
Bai 2018 [26]	Unclear	Unclear	High	Unclear	Unclear	Unclear	Unclear
Cheng AP, 2011 [23]	Unclear	Unclear	Unclear	Unclear	Unclear	Low	Unclear
Cui XX, 2015 [22]	Low	Unclear	Unclear	Unclear	Unclear	Low	Unclear
Guang, 2017 [25]	Low	Unclear	High	Unclear	Unclear	Unclear	Unclear
He F, 2013 [21]	Unclear	Unclear	High	High	Unclear	Low	Low
Liang, 2018 [27]	Low	Unclear	High	Unclear	Unclear	Unclear	Unclear
Liu SR, 2015 [20]	Low	Unclear	Unclear	Unclear	Unclear	Low	Unclear
Tang J 2012 [19]	Unclear	Unclear	Unclear	Unclear	Unclear	Low	Unclear
Wang WH, 2016 [18]	Unclear	Unclear	Unclear	Unclear	Low	Unclear	Unclear
Wen X, 2013 [8]	Low	Unclear	Unclear	Unclear	Unclear	Low	Unclear
Yang XQ, 2016 [17]	Low	Unclear	Unclear	Unclear	Unclear	Low	Unclear
Zhang QY, 2016 [16]	Low	Unclear	Unclear	Unclear	Unclear	Low	Unclear
Zhe, 2017 [24]	Low	Unclear	High	Unclear	Unclear	Unclear	Unclear
Acupressure							
Guo 2017 [32]	Low	Unclear	High	Unclear	Unclear	Unclear	Unclear
Huang 2018 [33]	Low	Unclear	High	Unclear	Unclear	Unclear	Unclear
Maa, 1997 [30]	Low	Unclear	Low	Unclear	Low	Unclear	Low
Tsay, 2005 [28]	Unclear	Unclear	Low	Low	Low	Unclear	Unclear
Wu, 2004 [31], 2007 [29]	Unclear	Unclear	Unclear	Unclear	Unclear	Unclear	Unclear
Wu, 2017 [34]	Low	Unclear	High	Unclear	Unclear	Unclear	Unclear
Xu 2018 [35]	Unclear	Unclear	High	Unclear	Unclear	Unclear	Unclear
Ear acupuncture							
Cao L, 2012 [36]	Low	Low	Low	Low	Low	Unclear	Unclear
Hu ZH, 1997 [41]	Unclear	Unclear	Unclear	Unclear	Low	Unclear	Unclear
Jin RF, 2009 [37]	Unclear	Unclear	Unclear	Unclear	Low	Low	Unclear
Li, 2017 [40]	Unclear	Unclear	High	Unclear	Low	Unclear	Unclear
Pang CL, 2014 [39]	Unclear	Unclear	Unclear	Unclear	Low	Unclear	Unclear
Pang CL, 2016 [38]	Unclear	Unclear	High	Unclear	Low	Low	Unclear
Acupressure plus ear acupuncture							
Rao, 2017 [42]	Low	Unclear	High	Unclear	Unclear	Unclear	Unclear
Cupping							
Xiao W, 2009 [43]	Unclear	Unclear	High	Unclear	Low	Unclear	Unclear

For AcuTENS trials, seven studies (100%) had a low risk of bias in “random sequence generation” and six (86%) in the “blinding of participants” (the other trial was considered unclear), however the most critical items were “allocation concealment” and “blinding of outcome assessment” with five (71%) and three (43%) trials classified as unclear due the lack of reporting. “Selective outcome reporting” was mainly classified as unclear (6 trials) due the fact that we could not find trials protocols.

We found an important lack of reporting in all trials and items for moxibustion, meaning that there was an unclear risk of bias for this technique.

Reporting was also poor for acupressure, and therefore the risk of bias was considered unclear. Low risk of bias was considered in only four trials (57%) for “random sequence generation”, none (0%) for “allocation concealment”, two trials (28%) for “blinding of participants” and one (14%) for “blinding of outcome assessment”.

Only one trial (16%) reported enough information for ear acupuncture to assess the risk of bias, which was classified low for “random sequence generation”, “allocation concealment”, “blinding of participants” and “blinding of outcome assessment”.

Regarding the trial combining acupressure and ear acupuncture, only the risk of bias for “random sequence generation” was classified as low.

The only trial included for cupping therapy, had an unclear risk of bias in all items except “incomplete outcome data” which was classified as having a low risk of bias.

### Synthesis of results

#### AcuTENS

##### AcuTENS vs. sham AcuTENS

*Dyspnoea*

**Fig. 2:**
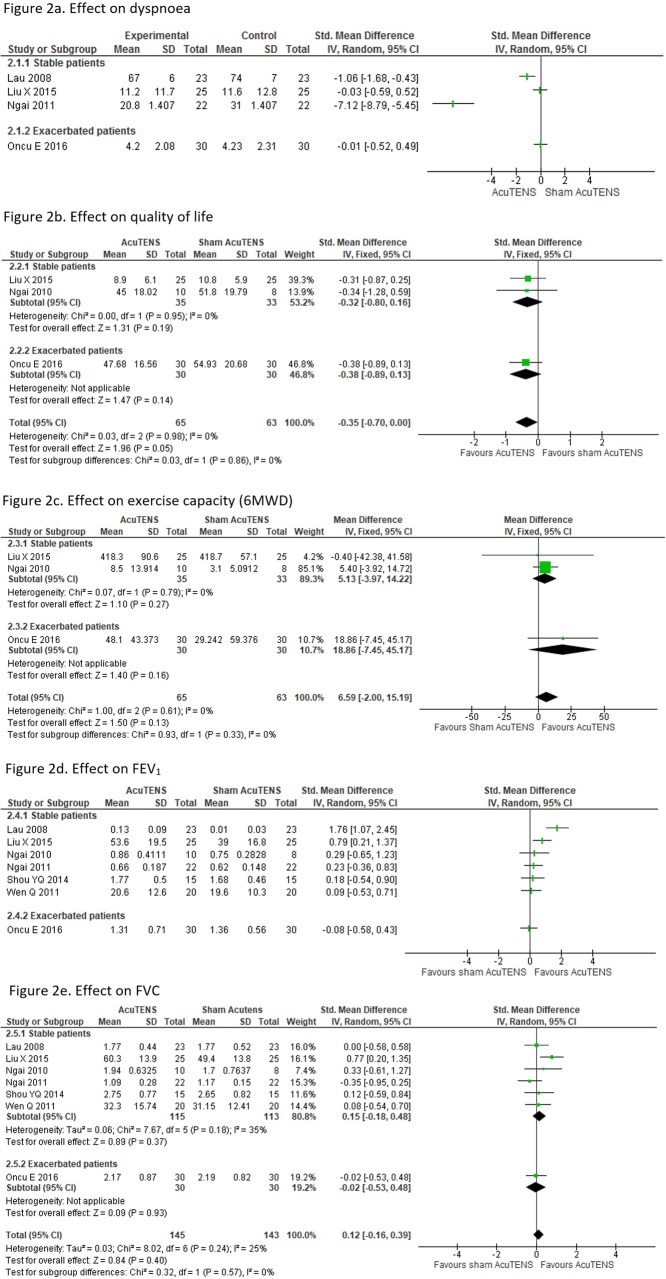
Meta-analysis of AcuTENS vs Sham

Four trials assessed dyspnoea, comparing AcuTENS vs sham AcuTENS, three included stable patients [9, 10, 12] and one included exacerbated patients [13]. The Dyspnoea Visual Analogue Scale (DVAS) was used in three trials [9, 10, 12], and the other one used the Borg Scale [13]. A meta-analysis could not be performed due to the high heterogeneity (I^2^ = 96%). In the three trials with stable participants, two showed an improvement in dyspnoea in a single session treatment [9, 12], and the other showed no difference between groups in a 4 week treatment [10]. The trial with exacerbated participants [13] did not show any effect compared with the sham intervention (Fig. 2a).

*Quality of life*

Three trials assessed QoL, two including stable patients [10, 11] and one with exacerbated patients [13]. QoL was assessed with the St Gorge’s Respiratory Questionnaire (SGRQ) in 2 trials [11, 13] and the COPD Assessment Test (CAT) in the other one [10]. Meta-analysis of all three trials (128 participants) did not show statistical differences between real and sham AcuTENS (Std. MD: -0.35 95%CI: − 0.70, 0.00 I^2^ = 0%) (Fig. 2b).

*Exercise capacity*

Three trials assessed exercise capacity, two with stable patients [10, 11] and one including exacerbated patients [13]. All trials used the six minutes walking distance test (6MWD). Meta-analysis of the three trials (128 participants) did not show a statistical improvement between AcuTENS and Sham (6MWD MD: 6.59 95%CI: − 2.00, 15.19 I^2^ = 0%) (Fig. 2c).

*Lung function*

Lung function (FEV_1_ and FVC) was assessed in seven trials, six including stable patients [9–12, 14, 15] and one with exacerbated participants [13]. Meta-analysis was not possible for FEV_1_ due heterogeneity (I^2^ = 73%), even in the subgroup analysis (stable subgroup *I*^*2*^ = 71%). Of the seven trials, two [9, 10] showed statistic benefit for AcuTENS and five other trials indicated no difference between groups [11–15] (Fig. 2d). Meta-analysis of the seven trials [9–15] (288 participants) for FVC showed no benefit for the AcuTENS group (Std. MD: 0.12 95%CI: − 0.16, 0.39 *I*^*2*^ = 25%) (Fig. 2e).

ADVERSE EVENTS

Only two trials attempted to report adverse events, Shou [14] reported that the technique was safe and Ngai [12] reported no associated adverse effects.

### MOXIBUSTION

#### Moxibustion vs. no moxibustion

##### Dyspnoea

Only one trial assessed dyspnoea [25]. In this trial authors reported a greater reduction on the mMRC scale in the moxibustion group compared with the control after a 12 week intervention (MD: − 1.70, Sd: 0.47 vs MD: − 1.03, Sd: 0.18, *p* < 0.05).

##### Quality of life

**Fig. 3:**
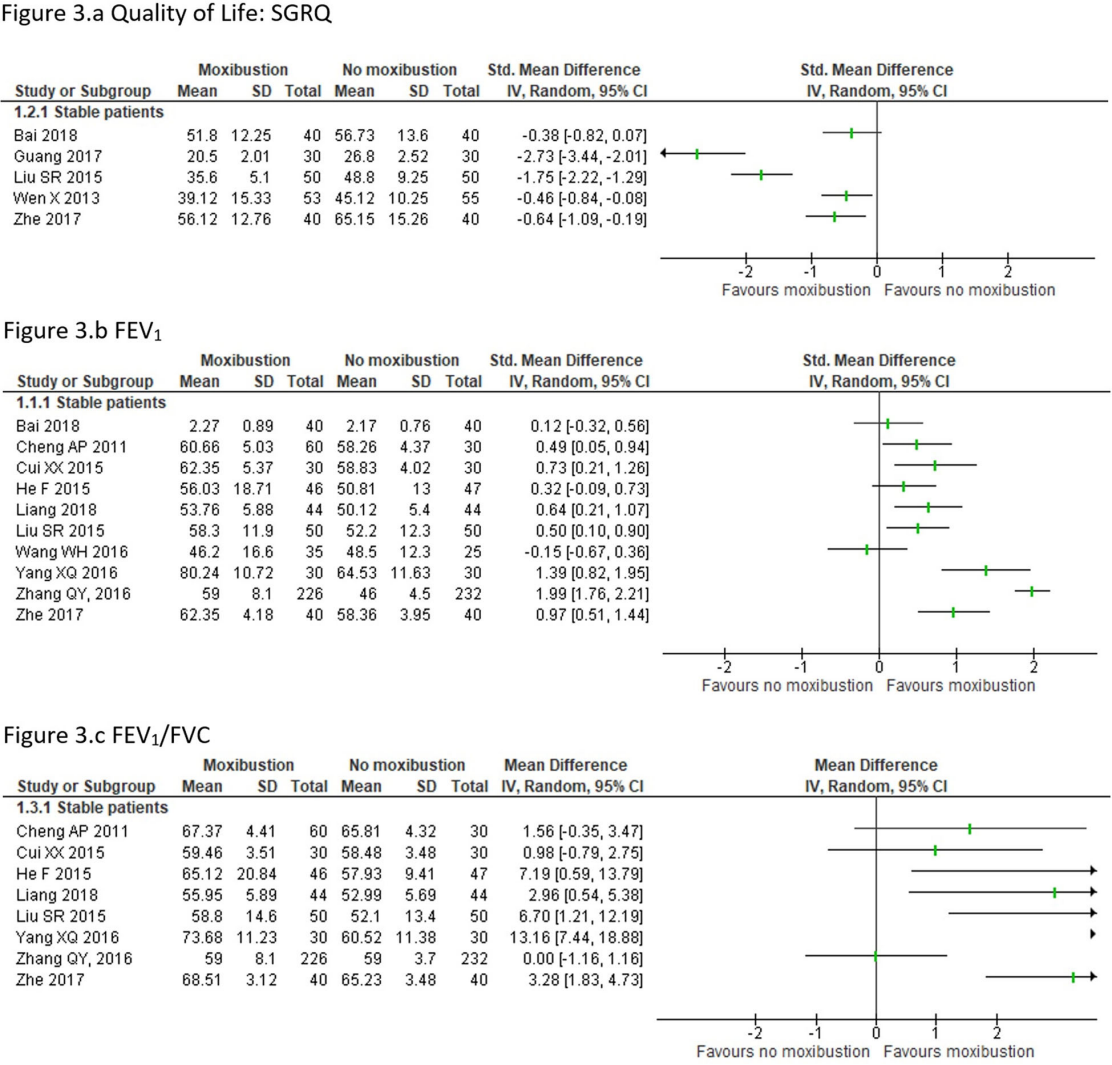
Meta-analysis of Moxibustion vs no Moxibustion

Five trials assessed QoL, four using the SGRQ [8, 20, 24, 26] and one using the CAT [25]. One trial did not report total scores of the SGRQ but only scores from each component separately [20], we calculated total scores using that data. Meta-analysis was not possible due the great heterogeneity (I^2^ = 92%). Out of five trials, four showed an improvement in QoL in the moxibustion group [8, 20, 24, 25] while one trial did not observe statistical differences [26]. (Fig. 3a).

##### Exercise capacity

Only one trial studied the effect of moxibustion on exercise capacity [20]. In this trial with 100 participants the authors reported an improvement in this outcome using the 6MWD (t = 3.568, *p* < 0.001), but no difference between groups was reported.

##### Lung function

Ten trials analysed lung function, all using FEV_1_ [16–18, 20–24, 26, 27], eight using FEV_1_/FVC [16, 17, 20–24, 27] and three trials using FVC [18, 20, 21]. Meta-analysis was not possible due to the high heterogeneity for FEV_1_ and FEV_1_/FVC (I^2^ = 93 and 80% respectively). Seven trials for FEV_1_ showed a statistical benefit for moxibustion [16, 17, 20, 22–24, 27] and three showed no effect [18, 21, 26] (Fig. 3b). Five trials for FEV_1_/FVC showed benefits for moxibustion [17, 20, 21, 24, 27] and the other three did not show statistical differences [16, 22, 23] (Fig. 3c).

##### Adverse events

No trials reported adverse events.

### Acupressure technique

#### Acupressure vs. sham acupressure

##### Dyspnoea

Dyspnoea was assessed in 2 trials (3 publications) using VAS (2 trials) [29, 30], the modified Borg scale (1 trial) [25] and the Pulmonary Function Status and Dyspnoea Questionnaire-Modified (PFSDQ-M) (1 trial) [31]. Meta-analysis was not possible since the study by Maa [30] used a cross-over design. Wu et al. reported a statistical improvement in the real acupressure group using VAS (43.43 mm vs 48.97 mm *p* < 0.01) [29] and the dyspnoea subscale of the PFSDQ-M (MD: − 0.98, *p* < 0.01) [31]. Maa [30] reported a statistical improvement in the real acupressure group using the VAS (*p* = 0.009) but not with the Borg scale (*p* = 0.38).

##### Exercise capacity

Two trials, both including stable patients, studied the effect of acupressure on exercise capacity using the 6MWD [30, 31]. Again, due to the cross over design used by Maa [30], a meta-analysis was not performed. While Maa did not report statistical differences between groups (*p* = 0.67), Wu et al. [26] did report a statistical improvement in the acupressure group (*p* < 0.001), none of the trials reported mean differences between groups.

##### Anxiety

Two trials reported anxiety in stable patients [30, 31]. The scales used for measuring were the Spielberger’s State Anxiety Inventory (SSAI) [31] and the anxiety subscale of the Bronchitis-Emphysema Symptom Checklist (BESC) [30]. Meta-analysis was not possible due to the cross-over design of Maa et al. Wu et al. reported statistical improvement in the acupressure group compared with sham intervention in the SSAI (− 8.50 vs − 0.14, p < 0.01) [31]. Ma et al. reported a significant improvement in the acupressure group using the BESC (*p* < 0.05) but the mean difference between groups was not reported [30].

##### Depression

One trial [29], which included stable participants, reported a greater reduction on the Geriatric Depression Scale (GDS) in the acupressure group than the sham group (− 2.09 vs 0.14 p < 0.001).

#### Acupressure vs no acupressure

##### Dyspnoea

Only one trial, comparing the addition of acupressure to usual care with usual care alone in patients under mechanical ventilation, assessed dyspnoea [28]. The authors reported a statistical reduction in dyspnoea of 6.77 points on VAS scale (SE: 2.59, *p* = 0.009).

##### Quality of life

Three trials studied QoL in stable participants, with one trial using the SGRQ [32], one trial using the CAT scale [33] and one trial using the Generic Quality of Life Inventory-74 (GQOL-74) [34]. The trial from Wu et al. [34] could not be included in the meta-analysis since authors did not report the GQOL-74 global score. Meta-analysis of the other two trials showed a statistically significant improvement on QoL (SMD: -0.63 95%CI: − 0.88, − 0.39 I^2^ = 0%) (Supplementary material 2.a).

##### Exercise capacity

One trial assessed exercise capacity using the 6MWD in stable participants [33]. Authors reported a greater distance walked in the acupressure group compared with the control (MD: 384.38 m, SD: 21.08 vs MD: 370.00 m, SD:23.74, *p* = 0.010).

##### Anxiety

Tree trials assessed anxiety, with two including stable participants [32, 35] and one with exacerbated participants [28]. The scales used were the VAS [28] and the Hamilton Anxiety Rating Scale (HAM-A) [32, 35]. Meta-analysis if the two trials with stable participants (332 participants) showed a statistical reduction of 4.83 points in the HAM-A scale (95%CI: − 5.71, − 3.94 I^2^ = 0%) (Supplementary material 2.b). The trial including exacerbated participants did not report enough data to be included in the analysis but also reported a statistical improvement compared with the control group (VAS MD: -6.74, SE: 2.68, *p* = 0.011).

##### Depression

Only one trial including sable participants reported depression using the Hamilton Depression Rating Scale (HAM-D) [32]. In this trial authors reported lower depression levels in the experimental group after the intervention (MD: 12.4, SD:4.36 vs MD: 19.1, SD: 6.1, *p* < 0.05).

##### Lung function

Lung function was assessed in two trials with stable participants [32, 33], both trials reported no statistical differences between acupressure and control group in FEV_1_, FVC and FV_1_/FVC.

##### Adverse events

Only one trial considered the possibility of adverse events and reported no skin reactions at the areas where acupressure was applied [30].

### Ear acupunture

#### Ear acupuncture vs sham ear acupuncture

Only one trial, including 30 exacerbated participants, compared ear acupuncture vs sham [36]. In this trial the investigators found a significant improvement in quality of life and lung function (FEV_1_, FEV_1_/FVC) (*p* < 0.05) after a 20-day intervention. Data on the effect size was not reported.

#### Ear acupuncture vs no ear acupuncture

##### Dyspnoea

Only one trial including stable participants assessed dyspnoea, reporting a statistical reduction in the mMRC scale in the two ear acupuncture groups compared with the control [40].

##### Lung function

Four trials assessed lung function [37–40], all of them assessing FEV_1_ and FEV_1_/FVC. Meta-analysis (224 participants) showed no statistical difference for this comparison on FEV_1_ (MD: 0.05 L 95%CI: − 0.05, 0.14 I^2^ = 0%) (Supplementary material 3.a) and FEV_1_/FVC (MD: 1.03 95%CI: − 1.16, 3.22 I^2^ = 0%) (Supplementary material 3.b).

#### Ear acupuncture vs drugs

One trial compared a single intervention treatment with salbutamol inhalation in 32 exacerbated patients [41]. The authors found a significant improvement in FEV_1_ (t = 2.62, p < 0.05), and no difference in FVC (t = 0.34, *p* > 0.05).

##### Adverse events

No trials reported adverse events.

### Acupressure plus ear acupuncture

One trial analysed the effect of acupressure combined with ear acupuncture plus standard therapy compared with standard therapy alone in lung function [42]. This trial, which included 120 stable participants, reported a significant improvement in the intervention group in FEV_1_, FVC, FEV_1_/FVC compared with the control group (*p* < 0.05).

### Cupping

Only one trial with 60 participants studied the effect of cupping [43]. The authors reported an improvement in cough, expectoration, dyspnoea and wheezing (p < 0.05) using the TCM syndrome integral. Data on the effect size was not reported. No adverse events were reported.

## Discussion

This is the first systematic review that evaluates independently the effectiveness of the different non-filiform needle acupuncture techniques for COPD. For practical reasons we only analysed the techniques that are most commonly used outside China; AcuTENS, moxibustion, acupressure, ear acupuncture and cupping (33 trials).

Our results do not show strong evidence for any non-filiform acupuncture techniques, only acupressure seams to improve dyspnoea and anxiety, based in low quality trials. The low number of trials assessing important outcomes, the great heterogeneity and the small size of most of the studies implies that these results must be interpreted with caution.

Overall, only six meta-analysis could be performed, with only two of them showing positive results. Heterogeneity was a big issue, even using subgroups for stable and exacerbated participants. This issue was even greater for the moxibustion technique, with no possible meta-analysis from three comparisons. We also found that very few trials reported important clinical outcomes commonly assessed in COPD such as dyspnoea (7/33), QoL (11/33), exercise capacity (7/33), anxiety (5/33) and depression (2/33). No important adverse events were reported for any technique.

Dyspnoea was improved in all 3 acupressure trials (2 trials vs sham and 1 trial vs no acupressure). For the other non-filiform acupuncture techniques only 1 trial using ear acupuncture and 2 trials using AcuTENS had positive results on dyspnoea, remarkably, both studies used a single session treatment**.** Acupuncture techniques mainly target the stimulation of cutaneous and muscular afferent fibres which lead to the stimulation of many brain nuclei networks, leading to the release of opioid peptides [44]. This mechanism has been usually accepted to explain acupuncture analgesic effects, but could also be used to explain acupuncture effects on dyspnoea, since endogenous opioids modulate dyspnoea in patients with COPD [45]. This mechanism has been better studied for the AcuTENS technique, which has been shown to increase B-endorphin levels which correlate with respiratory rate reduction [12]. However, improvements on dyspnoea were only seen for AcuTENS in single session trials but not in longer trials.

Quality of life is one of the main patient-related outcomes in clinical trials, however it was only studied in 11 trials. While meta-analysis of the 3 AcuTENS trials showed a tendency for improvement, results were not statistically significant. Quality of life was improved in 5 moxibustion trials with stable participants (2 vs sham and 3 vs no acupressure) and one ear acupuncture trial with exacerbated participants, all low-quality trials. It is to note that bowth trials comparing acupressure with sham acupressure lasted from 14 to 20 days and while seeing those changes in exacerbated patients in such a short time seems reasonable, it is quite surprising to find them in stable participants.

Exercise capacity is an important marker that results from a range of effects of COPD. Statistical differences in the 6MWD were not seen in the meta-analysis of the 3 AcuTENS trials however, while in the two trials with stable participates differences between groups were inexistent, in the trial with exacerbated participants an improvement was observed although CI was too wide to show statistical significance. One moxibustion trial and two acupressure trials reported benefits while another acupressure trial did not, but only one did report data of differences between groups.

Anxiety is strongly related to dyspnoea perception in COPD since the lack of breath is one of the most limiting symptoms experimented by patients. This correlation is seen in acupressure trials were all trials showing improvement on anxiety (5 trials) and dyspnoea (3 trials). Surprisingly this outcome was not studied in any other technique.

Finally, the effects on pulmonary function should be taken with caution. The heterogeneity of the results and the unexpected changes because the chronicity of the disease, prevent to any deeply interpretation. This outcome has been the most studied in the included trials (25/33), however, since pulmonary function is not expected to improve in COPD, no matter what treatment is used, we think future trials should not consider it as one of the main outcomes.

Coyle et al. [46] and Wang et al. [37] previously examined the effect of different acupuncture techniques for COPD. Coyle reviewed 16 trials (published between 1995 and 2007) of all kinds of acupuncture interventions, including non-pharmacological (filiform needle, moxibustion and acupressure, etc.) and pharmacological (herb plasters on acupuncture points). They concluded that acupuncture was beneficial for COPD patients in outcomes like dyspnoea, exercise capacity and quality of life, however evidence was low due to the methodological flaws of the included studies. Moreover, they plotted together all acupuncture techniques which might have caused some bias in the results. Wang’s review included 19 trials and concluded that acupuncture might be effective in improving functional effects and quality of life in COPD patients. However, this review only included acupuncture techniques such as manual acupuncture, warm acupuncture, electroacupuncture and ear acupuncture, but all other non-invasive technique such as single moxibustion, acupressure or acuTENS were excluded.

This review has several limitations. First, due to the great heterogeneity between trials, only seven meta-analyses could be performed. Secondly, important clinical outcomes for COPD, such as dyspnoea, QoL, exercise capacity, anxiety and depression were only studied in a small number of trials, reducing the number of trials that could be combined in each meta-analysis. Combined with the first point, this led to a low inspection efficiency of the results. Third, the trials included had inadequate reporting, especially for random sequence generation and allocation concealment, meaning that they had an uncertain risk of bias. Fourth, although no important adverse events were reported, this outcome has not been systematically explored. Fifth, it is difficult to extrapolate these results for different populations since, except one, all trials were performed in China. Finally, we identified other non-pharmacological acupuncture interventions in this review, such as catgut implant, thick needle, acupoint incision, wet cupping, floating needle and intradermal needle, however, due to the rare use of those techniques outside China, and the complexity of the review, we did not include them in this review analysis.

## Conclusions

No strong evidence was found for any of the included outcomes for patients with COPD treated with non-filiform needle acupuncture techniques. Acupressure could improve dyspnoea, quality of life and anxiety, but this is only based on low quality trials.

Evidence is very low in this review due to the unclear risk of bias in the trials included, and the great heterogeneity between them. Further studies should include main outcomes for COPD assessment such as dyspnoea, quality of life, exercise capacity and anxiety since we found many studies mainly targeting pulmonary function. Well-designed trials are needed to elucidate its possible role in the treatment of COPD.

## Supplementary information

**Additional file 1.** Supplementary material 1: Search strategies.

**Additional file 2.** Supplementary material 2: Meta-analysis of Acupressure vs no Acupressure.

**Additional file 3.** Supplementary material 3: Meta-analysis of Ear acupuncture vs no Ear acupuncture.

## Data Availability

All data generated or analysed during this study are included in this published article and its supplementary information files. During the preparation of this paper Carles Fernández was also given a grant from the Spanish Education Ministry.
