# Supplementary material for: Acupuncture techniques for COPD: a systematic review
Source: BMC Complement Med Ther. 2020 May 6;20:138. doi: 10.1186/s12906-020-02899-3 (PMC7323612; doi:10.1186/s12906-020-02899-3)
Supplement: Supplementary file 1 — Additional file 1. Supplementary material 1: Search strategies. [file 12906_2020_2899_MOESM1_ESM.docx]

# Supplementary material 1: Search strategies for each database

Search strategy for CENTRAL and MEDLINE databases:

1- "Acupuncture Therapy" (Mesh)

2- Acupuncture (Mesh)

3- "Acupuncture, Ear" (Mesh)

4- "Acupuncture, points"(Mesh)

5- Electroacupuncture (Mesh) or electro-acupuncture (tw)

6- Acu-TENS (tw) or "AcuTENS" (tw)

7- Moxibustion (Mesh)

8- Acupressure (Mesh)

9- Acup*(tw)

10- Or/1-9

11- COPD (Mesh)

12-"Pulmonary Disease, Chronic obstructive" (Mesh)

13- Bronchitis (Mesh)

14- "Obstructi*lung disease" (tw)

15- "Obstructi*airway disease" (tw)

16- Emphysema (Mesh) or "Pulmonary Emphysema" (Mesh)

17- "Chronic Air flow Limitation" (tw)

18- "Chronic Air flow obstruction" (tw)

19- "Chronic respiratory disease" (tw)

20- Or/11-19

21- 10 And 20

22- Randomized controlled trials (pt)

23- Controlled Clinical Trials (pt)

24- Randomized (tiab) or Randomly (tiab)

25- Placebo (tiab)

26- Trial (tiab)

27- Or/22-26

28- 21 And 27

Search strategy for Pedro database:

Therapy: Acupuncture

Topic: Chronic respiratory disease

Method: Clinical trial

Search strategy for Psycinfo database:

(Any Field:(COPD) OR Any Field:(Chronic obstructive pulmonary disease) OR Any Field:(Obstructi* lung disease) OR Any Field:(bronchitis) OR Any Field:(obstruci* airway disease) OR Any Field:(emphysema) OR Any Field:(pulmonary emphysema) OR Any Field:(chronic airflow limitation) OR Any Field:(chronic airflow obstruction) OR Any Field:(chronic respiratory disease) AND (Methodology:("Treatment Outcome"))) AND (Any

Field:(acupuncture) OR Any Field:(electroacupuncture) OR Any Field:(AcuTENS) OR Any Field:(moxibustion) OR Any Field:(acupressure) OR Any Field:(acup*)

Search strategy for Ovid EMBASE database:

1 exp acupuncture

2 acup*.tw.

3 exp electroacupuncture/

4 electro?ac*.tw.

5 acu-TENS.tw.

6 exp acupressure/

7 exp moxibustion/

8 moxibustion.tw.

9 1 or 2 or 3 or 4 or 5 or 6 or 7 or 8

10 exp chronic obstructive lung disease/

11 exp bronchitis/

12 obstructive lung disease.tw.

13 obstructive airways disease.tw.

14 exp lung emphysema/

15 chronic airflow limitation.tw.

16 chronic airflow obstruction.tw.

17 chronic respiratory disease.tw.

18 10 or 11 or 12 or 13 or 14 or 15 or 16 or 17

19 9 and 18

Search strategy for Ovid AMED database:

1 exp Acupuncture/

2 acup*.tw.

3 exp Electroacupuncture/

4 exp acupuncture therapy/

5 electro?ac*.tw.

6 acu-TENS.tw.

7 moxibustion.tw.

8 1 or 2 or 3 or 4 or 5 or 6 or 7

9 exp Lung diseases obstructive/

10 exp Bronchitis/

11 obstructive lung disease.tw.

12 obstructive airways disease.tw.

13 exp Emphysema/

14 chronic airflow limitation.tw.

15 chronic airflow obstruction.tw.

16 chronic respiratory disease.tw.

17 9 or 10 or 11 or 12 or 13 or 14 or 15 or 16

18 8 and 17

Search strategy for CINAHL dabase:

S1(MH "Acupuncture+")

S2(MH "Electroacupuncture")

S3(MH "Moxibustion")

S4(TX acup*)

S5(TX electro acup*)

S6(TX acu TENS*)

S7(TX moxibustion)

S8 S1 OR S2 OR S3 OR S4 OR S5 OR S6 OR S7

S9(MH "Pulmonary Disease, Chronic Obstructive+")

S10 (MH "Bronchitis+")

S11 (MH "Emphysema+")

S12 (TX obstructive lung disease)

S13 (TX obstructive airways disease)

S14 (TX chronic airflow limitation)

S15 (TX chronic airflow obstruction)

S16 (TX chronic respiratory disease)

S17 S9 OR S10 OR S11 OR S12 OR S13 OR S14 OR S15 OR S16

S18 S8 AND S17

CNKI

(SU=慢性阻塞性OR SU=慢阻肺OR SU=COPD) AND (SU=针灸OR SU=针刺OR SU=针OR SU=穴OR SU=灸OR SU=红外OR SU=埋线OR SU= 拔罐OR SU=耳针OR SU=耳穴OR SU=小针刀OR SU=温针OR SU=腕踝针OR SU=头针OR SU=挑治OR SU=梅花针OR SU=七星针OR SU=皮肤针OR SU=激光针OR SU=火针OR SU=锋钩针OR SU=磁针OR SU=鼻针OR SU=蜂针OR SU=腹针) AND FT=随机万方(主题=慢性阻塞性OR 主题=慢阻肺OR 主题=COPD) AND (主题=针灸OR 主题=针刺OR 主题=针OR 主题=穴OR 主题=灸OR 主题=红外OR 主题=埋线OR 主题= 拔罐OR 主题=耳针OR 主题=耳穴OR 主题=小针刀OR 主题=温针OR 主题=腕踝针OR 主题=头针OR 主题=挑治OR 主题=梅花针OR 主题=七星针OR 主题=皮肤针OR 主题=激光针OR 主题=火针OR 主题=锋钩针OR 主题=磁针OR 主题=鼻针OR 主题=蜂针OR 主题=腹针) AND 全文=随机

VIP

(M=慢性阻塞性+ M=慢阻肺+M=COPD+ R=慢性阻塞性+ R=慢阻肺+ R=COPD )*(M=针灸+ M=针刺+ M=针+ M=穴+M=灸+ M=红外+ M=埋线+ M=拔罐+M=耳针+ M=耳穴+ M=小针刀+ M=温针+ M=腕踝针+ M=头针+ M=挑治+ M=梅花针+ M=七星针+ M=皮肤针+ M=激光针+ R=针灸+ R=针刺+ R=针+ R=穴+ R=灸+ R=红外+R=埋线+ R=拔罐+ R=耳针+ R=耳穴+ R=小针刀+ R=温针+ R=腕踝针+ R=头针+ R=挑治+ R=梅花针+ R=七星针+ R=皮肤针+ R=激光针+ R=火针+ R=锋钩针+ R=磁针+ R=鼻针+ R=蜂针+ R=腹针)* U=随机

CBM

(((("慢性阻塞性"[摘要:智能]) OR "慢阻肺"[摘要:智能]) OR "COPD"[摘要:智能]) AND ((((("针灸"[摘要:智能]) OR "针刺"[摘要:智能]) OR "针"[摘要:智能]) OR "埋线"[摘要:智能]) OR "拔罐"[摘要:智能]) OR "穴"[摘要:智能]) OR "灸"[摘要:智能])OR "红外"[摘要:智能]) AND "随机"[全字段:智能]
