# Supplementary figures and images for: Acupuncture techniques for COPD: a systematic review
Source: BMC Complement Med Ther. 2020 May 6;20:138. doi: 10.1186/s12906-020-02899-3 (PMC7323612; doi:10.1186/s12906-020-02899-3)

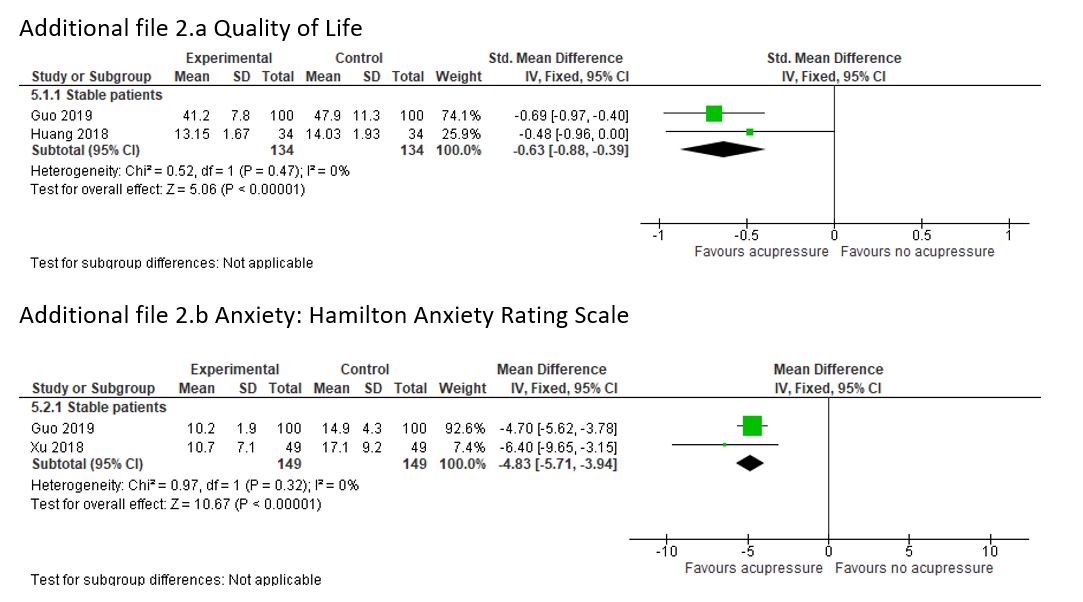

Supplement: Supplementary file 2 — Additional file 2. Supplementary material 2: Meta-analysis of Acupressure vs no Acupressure. [file 12906_2020_2899_MOESM2_ESM.jpg]

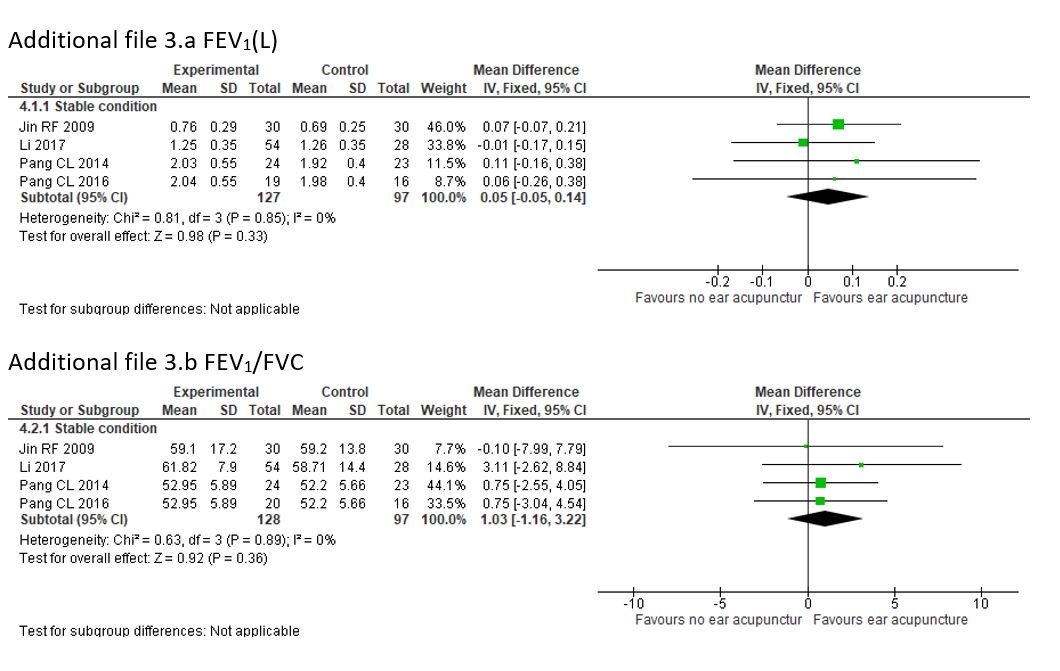

Supplement: Supplementary file 3 — Additional file 3. Supplementary material 3: Meta-analysis of Ear acupuncture vs no Ear acupuncture. [file 12906_2020_2899_MOESM3_ESM.jpg]
